# IgG response to *Mycobacterium tuberculosis* non-polar lipids and sonicated extracts among tuberculous meningitis patients

**DOI:** 10.1099/acmi.0.000131

**Published:** 2020-05-11

**Authors:** Prashant Giribhattanavar, Chris Pirson, Kavitha Kumar, Manaf Al-Qahtani, Ravi Shankar, Nagarathna Chandrashekar, Shripad Patil

**Affiliations:** ^1^​ Department of Neuromicrobiology, National Institute of Mental Health and Neuro Sciences (NIMHANS), Bangalore 560029, Karnataka, India; ^2^​ TB Research Group, Animal and Plant Health Agency, New Haw, Addlestone, Surrey, KT15 3NB, UK; ^3^​ Department of Medicine, Royal College of Surgeons in Ireland-University of Bahrain, Bahrain; ^4^​ Department of Biostatistics, National Institute of Mental Health and Neuro Sciences (NIMHANS), Bangalore 560029, Karnataka, India

**Keywords:** antimycobacterial antibodies, cerebrospinal fluid, ELISA, lipids, thin layer chromatography

## Abstract

**Introduction:**

The diagnosis of tuberculous meningitis (TBM) is a major global health concern due to its protean nature. There is a need to identify better biomarkers for the rapid and definitive diagnosis of TBM. Lipids have been poorly explored as diagnostic markers in TBM.

**Aim:**

Non-polar lipids (NPL) and mycobacterial sonicate extract (MTSE) antigens were assessed for diagnosis of *Mycobacterium tuberculosis *.

**Methodology:**

A total of 110 cerebrospinal fluid samples were categorized as confirmed, suspected and non-TBM cases according to clinical presentation and laboratory investigations, which were further analysed by NPL and MTSE ELISA.

**Results:**

The sensitivity and specificity of the NPL ELISA were 39.6 and 96 %, respectively, whereas the MTSE ELISA was 17 % sensitive and 92 % specific. The combination of the NPL and MTSE ELISA test was superior to these tests alone, with sensitivity and specificity of 43 and 88 %, respectively.

**Conclusion:**

This combination may be useful as an adjunct in the laboratory diagnosis of TBM. However, future studies in different settings among different populations, such as those with human immunodeficiency virus co-infection, are desirable to explore the full potential of biomarkers.

## Introduction

Tuberculosis (TB) was one of the top 10 cases of death globally in 2017, causing an estimated 1.3 million deaths. It was reported that 10.0 million people developed TB in 2017 [1]. Of the extra-pulmonary forms of TB, central nervous system TB (CNS-TB) is widely considered the most serious. According to initial reports, about 1 % of pulmonary TB cases develop into CNS-TB, which carries a high degree of mortality and morbidity [2]. CNS-TB commonly presents as tuberculous meningitis (TBM), tuberculoma or arachnoiditis. The protean nature of TBM makes its diagnosis challenging, often causing delays in diagnosis and leading to improper or inadequate treatment [3, 4]. Preliminary laboratory evidence is based on pleocytosis and altered levels of protein and glucose in the cerebrospinal fluid (CSF). However, these markers are non-specific [5]. Microbiological confirmation of TBM relies more on the detection of *Mycobacterium tuberculosis* (MTB) in the CSF by culture or smear techniques. However, microscopic examination lacks sensitivity due to low bacterial count in the CSF [6]. Conventional bacterial culture on solid media takes up to 8 weeks and lacks sensitivity. Different types of liquid culture techniques (MGIT, BACT/ALERT) are also used for the diagnosis of TBM. Nevertheless, these techniques are also time consuming (1–4 weeks for the result) and false negativity is difficult to rule out [7]. To overcome these diagnostic lacunae, a number of immunological and molecular methods have been explored [8]. A range of antigens have been tested for the immunodiagnosis of TBM including lipoarabinomannan, 14-kDa [9], A-60 [10], ESAT-6 [11] and MTB culture filtrate antigens. These antigens have a diverse range of sensitivity and specificity.

The mycobacterial cell wall is rich in lipid conjugates, and approximately 30 % of the mycobacterial genome codes for its cell wall components. Recent advances have highlighted the importance of lipids as immune modulators [12]. Papa *et al.* demonstrated the specificity of some lipids based on reaction to immune sera raised in rabbits with the corresponding antigen and with crude extracts of the MTB complex, which was non-reactive with 39 other mycobacterial species [13]. However, these lipids remain poorly explored as diagnostic markers in TBM. Hence, the present study aimed to extract non-polar lipids (NPL) from MTB H37Ra and to evaluate the NPL and MTB total sonicate extract (MTSE) as antigens for the immunodiagnosis of TBM.

## Methods

### Definition and classification of CSF samples

CSF samples from patients attending neurological services at the National Institute of Mental Health and Neurosciences (NIMHANS), Bangalore, were used in the present study after routine diagnosis.

A total of 110 CSF samples were included in the study, of which 48 were from male patients, with age ranging from 11 to 61 years and 62 female patients with age ranging from 18 to 66 years. Patients presenting with one or more of the clinical meningitis symptoms were included in the study (Table S1, available in the online version of this article). Human immunodeficiency virus (HIV)-positive cases were excluded from the study and non-TBM cases were included as controls.

Patients were categorized as one of the three groups as described by Marais *et al.* [14]: (i) patients whose CSF samples were culture-positive for MTB were taken as definite TBM cases (*n*=53); (ii) culture-negative samples from hydrocephalus patients, those showing neurological improvement to anti-TB treatment, or history of TB, and with a diagnostic score of 10–12 were considered as ‘probable’ TBM (*n*=11); and (iii) those diagnosed with TBM based on meningitis symptoms and preliminary laboratory evidence such as high cell count with a predominance of lymphocytes and with a diagnostic score of 6–9 were taken as ‘possible’ TBM cases (*n*=21). Probable and possible TBM were together considered as suspected TBM (*n*=32) cases. The suspected CSF samples were ruled out for other closely mimicking chronic meningitis. Cryptococcal infection was ruled out by India ink staining of CSF and inoculation on Sabouraud dextrose agar (SDA). Neurocysticercosis was ruled out by ELISA for detection of antibodies in the CSF. Other bacterial infections were ruled out by inoculating CSF in thioglycolate broth and incubation at 37 °C. Other fungal infections were ruled out by inoculating CSF onto SDA. Non-TBM samples were obtained from patients presenting with neurological complications but with a diagnosis other than TBM (*n*=25). These negative samples included pyogenic meningitis (*n*=12), Cryptococcal infection (*n*=6), neurocysticercosis (*n*=4) and non-infectious disease cases (*n*=3).

### Preparation of antigens

#### NPL

Extraction of NPL was performed as described previously [15]. Briefly, MTB grown in Middlebrook’s medium for 8 weeks was separated by centrifugation, and heat-killed cells were mixed with 20 ml of methanolic saline, and stirred for 15–20 min followed by petroleum ether (10 ml). The upper petroleum ether layer rich in lipids was separated and dried. The lipids was transferred to a pre-weighed glass tube in 4 : 1 CHCl_3_/CH_3_OH. Evaporation of the CHCl_3_/CH_3_OH was achieved using an nitrogen gas stream, and the tube was weighed to determine the net mass of NPLs. The NPL contents were analysed by two-dimensional TLC with the solvent system as per a previous report (Table S2) [15].

#### MTSE

MTSE antigen was prepared as described by Patil *et al.* [9]. Briefly, MTB H37Ra was grown for 8 weeks in Middlebrook’s medium. The culture was centrifuged and the pellet was resuspended 1:1 (w/v) in ice-cold PBS. The suspension was then agitated with glass beads in a homogenizer. The mixture was centrifuged at 14 000 *g* for 1 h at 4 °C. Protein concentration was measured using the Bradford method. The supernatant was aliquoted and stored at −20 °C until further use.

### ELISA

#### Coating of NPL

Coating of NPL was performed according to Julian *et al.* with slight modifications [16]. Briefly, NPL were dissolved in n-hexane and coated onto ELISA plates (Costar) at a concentration of 5 µg per well, which was air dried and then washed with Tris-buffered saline (TBS pH 7.5). The plates were subsequently blocked with 200 µl of 1 % TBS fat-free dried milk (Anik Spray) for 1 h.

#### Coating of MTSE

Coating of MTSE on ELISA plates was done according to Patil *et al.* [9]. Briefly, plates were coated (50 µl per well) with MTSE (10 mg ml^−1^) and incubated overnight followed by blocking with 1 % fat-free spray dried milk (Anik Spray) in PBS with 0.05 % Tween 20 (Sigma) for 1 h.

Fifty microlitres of diluted CSF (1 : 2 in TBS milk/PBST milk) was added in duplicate to the plates and incubated at ambient temperature for 1 h, and 50 µl of diluted mouse anti-human IgG (Dakopatts) was added as a conjugate (1 : 3000 in TBS milk) with washing in between. Finally, 75 µl per well of substrate was added [4 mg of OPD (Sigma) in 10 ml of phosphate citrate buffer containing 6 µl H_2_O_2_] and incubated for 10 min at room temperature. The reaction was stopped by adding 1 M H_2_SO_4_ (50 µl per well) and the OD was read at 492 nm using a plate reader (Magellan; Teccan).

### Data analysis

All continuous and categorical variables were summarized using appropriate measures for all four groups. To compare the characteristics between groups (confirmed vs. negative), the Mann–Whitney *U* test and Fisher’s exact test were used for continuous and categorical variables, respectively. Following standard definitions, a 2×2 contingency table and receiver operating characteristic (ROC) curve analysis were used to calculate all the diagnostic accuracy measures and confidence intervals. The Youden index was used to select cut-off points for OD values of NPL and MTSE. The accuracy measures were expressed as sensitivity, specificity, positive predictive value (PPV), negative predictive value (NPV), positive likelihood ratio (LR+) and negative likelihood ratio (LR-). Fleiss kappa (κ) statistics was used to assess the agreement between two tests, where a value of κ>0.75 is considered excellent, κ=0.40–0.75 is considered fair to good, and κ<0.40 is considered marginal to poor. A *P* value of <0.05 was considered statistically significant for proportional analyses. IBM SPSS Statistics for Windows, Version 22.0 was used for statistical analysis.

## Results

### Identification of NPL by 2D TLC

**Fig. 1.**
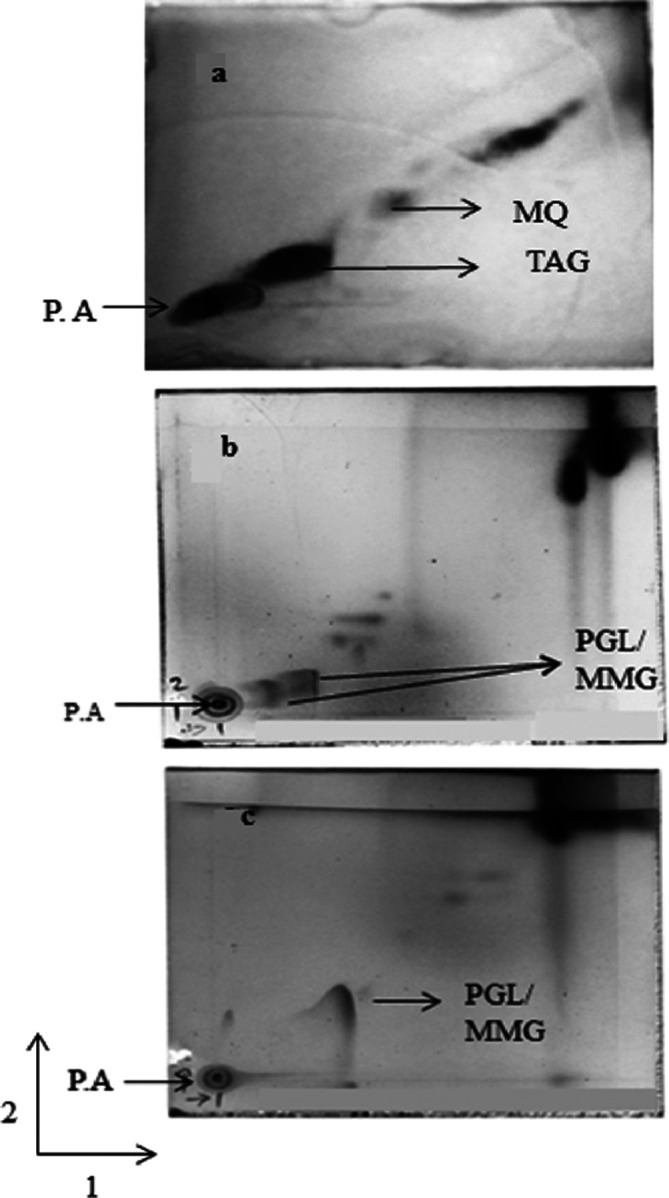
2D TLC analysis of NPL extracted from *M. tuberculosis *. The TLC plate was stained with phosphomolybdic acid. NPL analysed with (a) solvent system A, (b) system B and (c) system C.

Analysis of the NPL fraction using the least polar TLC system (Fig. 1a) identified the presence of menaquinone (MQ) and triacylglycerol (TAG). System B (Fig. 1b) revealed that the NPL fraction also contained phenolic glycolipids (PGL) and monomycolyl glycerol (MMG). System C (Fig. 1c) allowed further confirmation of both MMG and PGL.

### Performance outcomes of NPL and MTSE tests in the diagnosis of TBM

From the ROC curve generated using NPL ELISA data, the area under the curve (AUC) was 0.783 (SE=0.054, *P<*0.001) (Fig. S1). The Youden index revealed a cutoff of 0.19. At this cut-off, the sensitivity and specificity of the NPL ELISA were 69.8 % [95 % confidence interval (CI): 0.51–0.77] and 80 % (95 % CI: 0.64–0.95), respectively. Results of the NPL ELISA showed that 21 (39.6 %) out of 53 TBM-confirmed cases were positive. Among the 11 probable TBM cases, nine (81.81 %) were positive, and 10 (47.61 %) of the 21 possible TBM cases were positive; of the 25 negative controls, five showed reactivity.

By modifying the cut-off value to 0.38 to set it for maximum specificity while surrendering sensitivity (selection of rule-in test), the sensitivity and specificity of the assay were 39.6 % (95 % CI: 0.26–0.53) and 96 % (95 % CI: 0.88–1.00), respectively (Table 1). Of the 32 suspected TBM cases, 81.18 % (9/11) among probable TBM cases and 33.33 % (7/21) possible TBM cases were positive. Of the 25 negative controls, only one CSF of pyogenic meningitis (1/12) was reactive to NPLs at this cut-off value.

**Table 1.** Diagnostic power of NPL and/or MTSE ELISA

	Number of positive cases		Sensitivity (%) (95 % CI)	Specificity (%) (95 % CI)	PPV	NPV	Positive likelihood ratio	Negative likelihood ratio
	Confirmed TBM (*n*=53)	Non-TBM (*n*=25)						
Non-polar lipids	21	1	39.6 (0.26–0.53)	96 (0.88–1.00)	0.95 (0.87–1.00)	0.43 (0.30–0.56)	9.91 (1.41–69.55)	0.63 (0.50–0.79)
MTSE	9	2	17 (0.06–0.27)	92 (0.81–1.00)	0.82 (0.59–1.0)	0.34 (0.23–0.46)	2.12 (0.49–9.11)	0.90 (0.76–1.07)
Both non-polar lipids and MTSE	7	0	13 (0.04–0.22)	100 (1.0–1.0)	1 (1–1)	0.35 (0.24–0.46)	–	0.87 (0.78–0.96)
Either non-polar lipids or MTSE	23	3	43 (0.30–0.57)	88 (0.75–1.0)	0.88 (0.76–1.0)	0.42 (0.29–0.56)	3.62 (1.21–10.92)	0.64 (0.49–0.85)

CI, confidence interval; NPV, negative predictive value; PPV, positive predictive value; NPL: non-polar lipids; MTSE: mycobacterial sonicate extract

From the ROC curve generated using MTSE ELISA data, the AUC was 0.50 (SE 0.07, *P*=0.991) (Fig. S1). The Youden index revealed a cut-off of 0.61. At this cut-off, the sensitivity and specificity of the MTSE ELISA in patients with confirmed TBM and in non-TBM cases were 17 % (95 % CI: 0.06–0.27) and 92 % (95 % CI: 0.81–1.00), respectively, and it was found to be a good rule-in test for the diagnosis of TBM. With this cut-off, out of 53 confirmed TBM cases, nine were positive (16.98 %). None of the samples from probable TBM and possible TBM cases tested positive by MTSE ELISA. Among 25 non-TBM cases, only two pyogenic meningitis samples tested positive by MTSE ELISA (Table 1). There was moderate agreement between NPL and MTSE ELISA in the confirmed TBM group (κ=0.411). This agreement was poor between the non-TBM group (κ=0.042) (Table 2) and no agreement (κ=0) was noted in suspected cases.

**Table 2.** Diagnostic agreement between NPL and MTSE ELISA tests in confirmed TBM and non-TBM categories

Category	κ	95 % CI	SE
Confirmed TBM	0.411	0.155–0.666	0.13
Non-TBM	0.042	0.013–0.12	0.041

CI, confidence interval; κ, kappa statistic; SE, standard error.

### Combination of MTSE and NPL ELISA for the diagnosis of TBM

As shown in Table 1, NPL test sensitivity was significantly better than MTSE (*P*=0.020). However, there was no significant difference in the specificity of the tests (*P*=0.317). There was no significant difference between the NPV (*P*=0.309) and PPV (*P*=0.059) of the two tests. There was a significant difference between the MTSE test and the combined MTSE and NPL tests (*P*=0.0005). There was no significant difference between the NPL test and the combined MTSE and NPL tests (*P*=0.08). We also compared the detection of antibodies by both MTSE and NPL ELISA in the same patients and found that seven samples (13.2 %) were double positive in the confirmed TBM group and 22 (88 %) samples were double negative in the non-TBM group. In the confirmed TBM group, 13 (24.52 %) and two (3.77 %) were single positive by NPL and MTSE ELISA, respectively, and 30 (56.6 %) were double negative. In contrast, in the non-TBM group, one (4%) and two (8%) samples were single positive by NPL and MTSE ELISA, respectively. Interestingly, none of the samples tested double positive in the non-TBM group. Thus, it could be inferred that combining NPL ELISA with MTSE ELISA would improve the diagnostic accuracy for TBM.

**Fig. 2.**
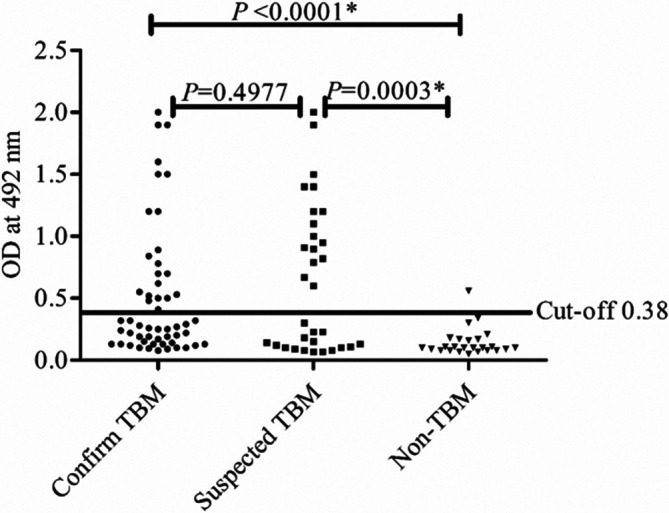
Scatter gram showing a comparison of OD values for the level-specific IgG to NPL antigen in CSF samples of confirmed TBM, suspected TBM and non-TBM patient groups. The horizontal line in the graph represents the cut-off value (0.38) obtained from the receiver operating curve. *P<0.05 was considered significant.

**Fig. 3.**
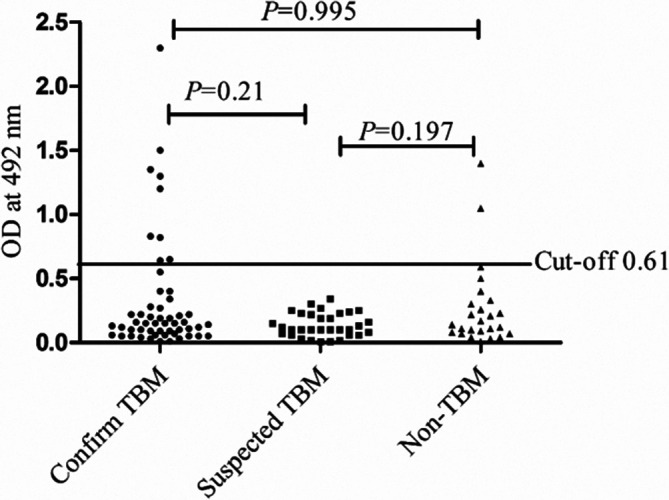
Scatter gram showing a comparison of OD values for the level-specific IgG to MTSE antigens in CSF samples of confirmed TBM, suspected TBM and non-TBM patient groups. The horizontal line in the graph represents the cut-off value (0.61) obtained from the Youden index calculator. *P<0.05 was considered significant.

In the present study, the presence of significantly high antibody levels against NPL in confirmed TBM (*P*<0.0001) and suspected TBM (*P*<0.0003) patients as compared to non-TBM cases was observed. There was no significant difference in NPL antibody levels between TBM and suspected TBM cases (*P*=0.497) (Fig. 2). Furthermore, in MTSE ELISA, there was no significant difference between the OD of confirmed TBM and non-TBM (*P*=0.99) cases; similarly, there was no difference between confirmed TBM cases and suspected TBM cases (*P*=0.21) and also between suspected TBM and non-TBM cases (*P*=0.197) (Fig. 3).

## Discussion

Our study has three important findings. First, the newly developed NPL-based ELISA technique for the diagnosis of TBM is suitable for low-resource settings. Second, we have shown the diagnostic strengths of the NPL antigen and routinely used MTSE antigen. Lastly, a multimodal approach is required for the diagnosis of TBM.

The mycobacterial cell wall is characterized by complex lipids, which are critical for the pathogenicity and immunomodulatory properties of the bacilli [17]. Extraction of mycobacterial lipids and their subsequent analysis by 2D TLC have been previously described [15]. In the present study, TLC analyses of the NPL extract identified characteristic mycobacterial lipids. Individual lipid spots such as MQ and TAG, characteristic PGL, and MMG obtained by TLC separation were found to be similar to those reported previously [15]. There has been a paucity of information regarding the application of NPLs of MTB for the immunodiagnosis of TBM. The present study showed NPLs were significantly immunogenic in TBM cases and showed 96 % specificity of the immune response. Furthermore, no cross-reactivity was noticed with clinically closely related conditions such as neurocysticercosis and neurocryptococcosis.

Many lipid antigens have been explored as markers for the diagnosis of TB. In one study, lipooligosaccharides, DAT and PGL were evaluated for the serodiagnosis of TB among children, giving 34 % sensitivity among culture-positive TB cases and 33 % sensitivity among culture-negative TB cases with 86 % specificity [18]. In another study with tuberculous glycolipid (TBGL), sensitivity for active TB patients was 81.1 % and specificity was 95.7 %. Sensitivity in patients with smear-negative and culture-negative active pulmonary TB was 73.5 % [19]. A recent study from China showed that TBGL is a component of the MTB cell wall, and anti-TBGL antibodies are used for the serodiagnosis of TB. Anti-TBGL IgG levels were measured in pulmonary TB (PTB) patients and extra-pulmonary TB (ETB) patients. Anti-TBGL IgG sensitivity was 68.9 and 46.2% in PTB and ETB, respectively, and specificity was 81.0 % [20]. However, little is known about the diagnostic utility of lipid antigens for the diagnosis of TBM.

One of the main causes of delayed diagnosis of TBM is the relatively mild symptoms of the disease. Thus, screening of patients with primary symptoms may be an important aspect to control TBM. Many tests have been developed apart from the gold standard (MTB culture), which is less sensitive (15 %) with CSF to diagnose TBM [6]. The liquid culture system MGIT has improved sensitivity, 27 % [7].

This is the first report on the utility of NPLs from MTB as regards diagnostic potential and compared with MTSE ELISA in TBM. NPL ELISA positivity was 39.6 % among confirmed TBM cases and 50 % among suspected TBM cases, and only one positive case among 25 non-TBM subjects was found. This finding is in concordance with our previous observation wherein the sensitivity of PGL-1 antigen ELISA in TBM patients is up to 42 % [21]. MTSE-based ELISA was positive in 16.98 % cases of confirmed TBM, 8 % among non-TBM cases and 0 % from suspected cases. Statistically there was moderate to poor agreement between NPL and MTSE tests among different categories of patients, which might be due to the heterogenic response from the host against MTB. This heterogeneity might be due to the variation in the immunogenetic background of the individual host or variation between the stage of the growth of MTB in the host and differential bacillary load in CSF. This also indicates the requirement for a multimodal approach for the diagnosis of TBM. Once these two tests were combined, positivity increased to 43.39 %. Using the above ELISA, three pyogenic meningitis cases (one by NPL and two from MTSE) were positive, which could not be confirmed for co-infection by mycobacteria. This ELISA is better than culture in terms of positivity, which will take up to 8 weeks to conclude. These immunoassays are robust compared to culture, which is the gold standard in the diagnosis of TBM.

Numerous studies have been reported on various other parameters as biomarkers in CSF for the diagnosis of TBM, including a predominance of lymphocytes, decreased glucose level and increased protein level [22]. Elevated CSF adenosine deaminase (ADA) activity has been observed in TBM patients [23]. However, these biochemical tests are general in nature, and not specific for TBM. Another automated technique based on real-time PCR is Xpert MTB/RIF, which also has a sensitivity of 48%, but was negative in all four patients tested in HIV-uninfected cases. Furthermore, it is poorly sensitive in suspected cases [24, 25]. The IFN gamma release assay (IGRA) is another technique that has been extensively used in the diagnosis of TB; for TBM, however, it is moderately sensitive according to meta-analysis and the authors also concluded that CSF PCR or ADA have better diagnostic accuracy than IGRAs in TBM [26].

Many researchers have also tried to detect specific antigens in CSF for serological diagnosis of TBM, such as ESAT-6, 14 kDa, MPT 63, 19 kDa, MPT64, 38 kDa, culture filtrate protein and LAM, with positivity ranging from 4.34 to 40 % [27–29]. In contrast to these reports, the combined ELISA showed high positivity in suspected TBM cases (50 %) and NPL ELISA was found to be a better diagnostic marker.

To our knowledge, this is the first report showing NPL as a biomarker in the diagnosis of TBM in conjunction with MTSE antigen. Additional studies are needed in different settings to assess the usefulness of this test as a diagnostic tool in different populations, especially where patients are immunosuppressed by HIV infection. The test also requires screening a large number of clinically suspected TBM cases. Future research is also needed to address the correlation of antibody levels in CSF with the outcome of anti-TB treatment and drug resistance.

## Supplementary Data

Supplementary material 1
