# Supplementary material for: IgG response to Mycobacterium tuberculosis non-polar lipids and sonicated extracts among tuberculous meningitis patients
Source: Access Microbiol. 2020 May 11;2(7):acmi000131. doi: 10.1099/acmi.0.000131 (PMC7497836; doi:10.1099/acmi.0.000131)

Supplementary table 1. The demographic and clinical characteristics of confirm and suspected TBM cases.

Variable	Confirmed TBM (n=53)	Probable TBM (n=11)	Possible TBM (n=21)	Non TBM* (n= 25)
Gender: Male	27(50.94%)†	4(36.36%)	11(52.38%)	12(48%)†
Age in years Mean ± SD (Range)	32.96 ±10.87 (11-57) ‡	36.9 ±11.57 (19-56)	42.28 ±12.78 (25-66)	37.52±10.83 (22-60) ‡
Presenting Symptoms	Number (Percentage; Range in days among whom symptoms were present)			
Headache	44 (83.01; 7-30)	8 (72.72; 5-60)	19 (90.47; 3-60)	-
Vomiting	8 (15.09; 1-2)	2 (18.18; 1-2)	2 (9.52; 1-2)	-
Fever	24 (45.28; 2-7)	5 (45.45; 2-10)	7 (33.33; 2-15)	-
Altered sensorium	14 (26.14; 2-6)	1 (9.09; 2)	12 (57.14; 1-7)	-
Neck stiffness	8 (15.09; 5-30)	1 (9.09; 60)	1 (4.76; 30)	-

†Fisher's Exact test between confirmed and non-TBM

‡Mann-Whitney *U* test between confirmed and non-TBM

*Clinical characteristics of non-TBM cases not assessed

Supplementary table 2 : Solvent system for separation of lipids

Solvent system	Run direction	Solvents	No. of runs	Lipids resolved
A	1<br>2	Petroleum ether : Ethyl acetate (98 : 2)<br>Petroleum ether : Acetone (98 : 2)	3<br>1	TAG, MQ
B	1<br>2	Petroleum ether : Acetone (92 : 8)<br>Toluene : Acetone (95 : 5)	3<br>1	AT, FA
C	1<br>2	Chloroform : Methanol (96 : 4)<br>Toluene : Acetone (80 : 20)	1<br>2	FA, GLY

TAG, triacyl glycerides; MQ, menaquinones; AT, acylated trehalose; FA, fatty acids; GLY, glycosides;

Supplementary Figure. 1. Receiver operating curve (ROC) showing sensitivity and specificity, constructed for IgG antibody response to *M. tuberculosis* non-polar lipid and *M. tuberculosis* sonicate extract (MTSE) antigens from TBM and non-TBM patients. Area under the curve non-polar lipid ELISA and MTSE ELISA were 0.78 (95%CI; 0.6776 -0.8892.*P*<0.0001. SE=0.053) and 0.5 (95%CI; 0.3630-0.6385. *P*=0.9915. SE=0.07) respectively. *P*< 0.05 considered significant. SE: Standard Error

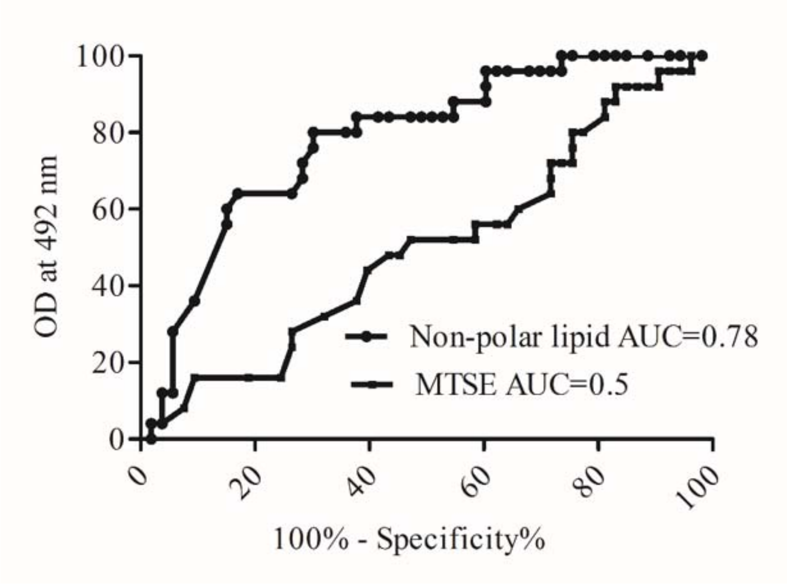

Supplement: Supplementary material 1 [file acmi-2-131-s001.pdf]
